# Construction of a New Growth References for China Based on Urban Chinese Children: Comparison with the WHO Growth Standards

**DOI:** 10.1371/journal.pone.0059569

**Published:** 2013-03-19

**Authors:** Xin-Nan Zong, Hui Li

**Affiliations:** Department of Growth and Development, Capital Institute of Pediatrics, Beijing, China; Indiana University, United States of America

## Abstract

**Introduction:**

Growth references for Chinese children should be updated due to the positive secular growth trends and the progress of the smoothing techniques. Human growth differs among the various ethnic groups, so comparison of the China references with the WHO standards helps to understand such differences.

**Methods:**

The China references, including weight, length/height, head circumference, weight-for-length/height and body mass index (BMI) aged 0–18 years, were constructed based on 69,760 urban infants and preschool children under 7 years and 24,542 urban school children aged 6–20 years derived from two cross-sectional national surveys. The Cole’s LMS method is employed for smoothing the growth curves.

**Results:**

The merged data sets resulted in a smooth transition at age 6–7 years and continuity of curves from 0 to 18 years. Varying differences were found on the empirical standard deviation (SD) curves in each indicator at nearly all ages between China and WHO. The most noticeable differences occurred in genders, final height and boundary centiles curves. Chinese boys’ weight is strikingly heavier than that of the WHO at age 6–10 years. The height is taller than that of the WHO for boys below 15 years and for girls below 13, but is significantly lower when boys over 15 years and girls over 13. BMI is generally higher than that of the WHO for boys at age 6–16 years but appreciably lower for girls at 3–18 years.

**Conclusions:**

The differences between China and WHO are mainly caused by the reference populations of different ethnic backgrounds. For practitioners, the choices of the standards/references depend on the population to be assessed and the purpose of the study. The new China references could be applied to facilitate the standardization assessment of growth and nutrition for Chinese children and adolescents in clinical pediatric and public health.

## Introduction

Growth charts are widely used as an essential tool for assessing the growth and nutritional status of the population and individual child. Considering the racial/ethnic diversity and environmental forces in human growth around the world [1]–[3], many countries [4]–[7] had therefore established their own growth references.

China started the 1^st^ National Survey on the Physical Growth and Development of Children in the Nine Cities of China (NSPGDC) in 1975 [8], and the 2^nd^, 3^rd^ and 4^th^ surveys were performed using the same study designs in the same 9 cities in 1985, 1995 and 2005, respectively [9]. This series of surveys was, so far, the largest nationally representative sample of infants and preschool children under 7 years in China. The Chinese National Survey on Student’s Constitution and Health (CNSSCH) had been carried out every 5 years since 1985 with a total of 5 times by 2005, covering all the 31 provinces, autonomous regions and municipalities [10]. This series of surveys was the largest nationally representative sample of school-age children and adolescents from 6 to 22 years. Data obtained from the two series of surveys was commonly expressed as observed means/SD and observed percentiles by the crude age groups in tabulated forms which could be inefficiently used in health care, clinical practice and research work.

In April 2006 the World Health Organization (WHO) released new standards for children from birth to 5 year [11], which were based on a population-based study conducted between 1997 and 2003 in Brazil, Ghana, India, Norway, Oman and the USA. Then in September 2007 the WHO published a growth reference for children and adolescents from 5 to 19 years, using the same sample of the 1977 National Center for Health Statistics (NCHS)/WHO growth reference [12].

In China, the existed growth reference values generated from the two series only covered incomplete age ranges [13]–[14], meanwhile, most data covering complete age ranges were based on local growth surveys in single city [15]–[18]. Considering the rapid secular growth trend in China in the past decades [9], [19], it was imperative to establish a set of new, smoothed, standardized growth curves for Chinese children and adolescents, based on up-to-date national data from the 4^th^ NSPGDC [20] and the 5^th^ CNSSCH [21], and the smoothing technique of the Cole’s LMS method widely used in many countries [22].

Here, we try to construct percentile and Z-score reference values for weight-for-age, length/height-for-age, head circumference-for-age, weight-for-length/height and body mass index (BMI)-for-age for Chinese children and adolescents aged 0–18 years and compare the differences of each indicator by gender between the China and WHO curves.

## Methods

### Data and Subjects

**Figure 1:**
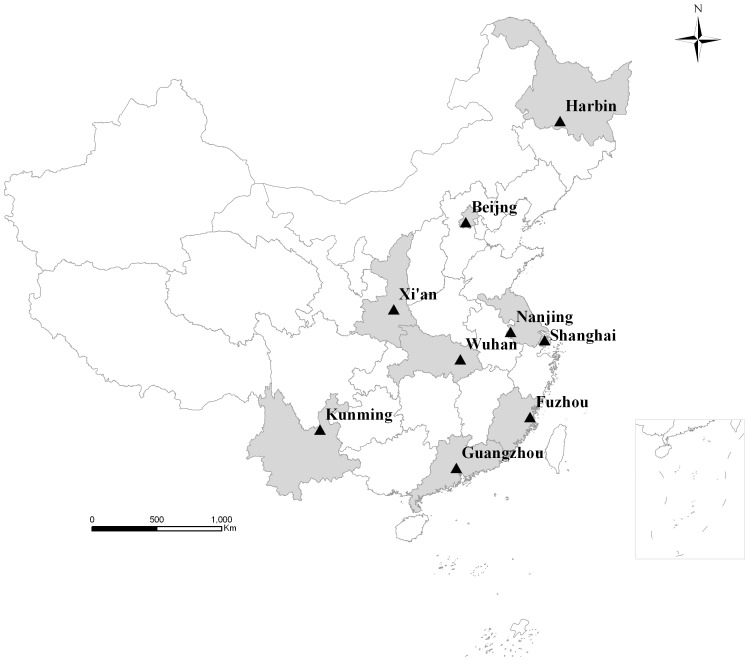
Geographical distribution of the 9 cities (Shaded their corresponding provinces) in China.

Children from 0 to 7 years: The 4^th^ NSPGDC was performed in 9 major cities of China between May and October 2005. Among the 9 cities, Beijing and Shanghai are municipalities, and the other seven are provincial capital cities, including Harbin, Xi’an, Nanjing, Wuhan, Guangzhou, Fuzhou, and Kunming (**Fig. 1**). Children under 7 years were divided into 22 age groups at an empirical interval. 150–200 subjects were recruited for each age group with different gender for urban/suburban of each city. Multistage stratified cluster sampling was used according to urban/suburban areas, districts, street community (children <3 years) and kindergarten (3–7 years). A total of 69,760 subjects with 34,901 boys and 34,859 girls (**Table 1**) were gathered from urban areas of the 9 cities. Exclusion criteria included temporary residents, history of premature birth, birth weight less than 2500 g, acute illness within a month, chronic illness, obviously malnourished and physically handicap.

**Table 1:** Numbers at each age group contributing to the LMS analyses, by gender.

The 4^th^ NSPGDC			The 5^th^ CNSSCH		
Age	Boys	Girls	Age	Boys	Girls
0–3 days	1554	1512	6 yrs-	784	795
1 mos-	1599	1573	7 yrs-	824	882
2 mos-	1571	1559	8 yrs-	846	869
3 mos-	1566	1588	9 yrs-	868	880
4 mos-	1589	1581	10 yrs-	878	876
5 mos-	1576	1580	11 yrs-	873	843
6 mos-	1604	1585	12 yrs-	889	870
8 mos-	1608	1622	13 yrs-	872	871
10 mos-	1584	1581	14 yrs-	873	869
12 mos-	1591	1595	15 yrs-	910	897
15 mos-	1583	1578	16 yrs-	875	872
18 mos-	1582	1572	17 yrs-	865	868
21 mos-	1582	1573	18 yrs-	792	778
2.0 yrs-	1580	1582	19–20 yrs	1039	1184
2.5 yrs-	1551	1561	Total	12188	12354
3.0 yrs-	1586	1601			
3.5 yrs-	1588	1600			
4.0 yrs-	1588	1602			
4.5 yrs-	1593	1599			
5.0 yrs-	1592	1595			
5.5 yrs-	1605	1597			
6–7 yrs	1629	1623			
Total	34901	34859			

Children from 6–20 years: The 5^th^ CNSSCH was conducted in 31 provinces of China between May and July 2005. Multistage stratified cluster sampling was used according to provinces/cities, socioeconomic classes, urban/rural areas, school, class. Each yearly age group consisted of 100 boys and 100 girls for urban/rural of each city/province. A total of 24,542 subjects with 12,188 boys and 12,354 girls (**Table 1**) were naturally selected from urban areas of Beijing, Shanghai and 7 provincial capital cities mentioned above (**Fig. 1**).

Data from urban areas of the 4^th^ NSPGDC (0–7 years) was merged with the data from urban areas of the corresponding 9 provinces/cities of the 5^th^ CNSSCH (6–20 years) to smooth the transition and the continuity at the entire age ranges between the two samples.

The NSPGDC and CNSSCH protocol studies were approved by the Local Institutional Review Boards, Ethics Committees of the Capital Institute of Pediatrics, and the School of Public Health of the Peking University respectively. Data aged 0–7 years from the NSPGDC was gathered through the child health care system constructed in 1970s while data aged 6–20 years from the CNSSCH through the school health monitoring network. In the two surveys, informed consent was verbal, but there was not ethics issue in the present study. Before the survey, the related issues including the purposes and procedures of the surveys, and the risk/discomforts and benefits of the surveys, were adequately explained by the staff of the cooperating groups to the parents, guardians, teachers and students involved in the surveys. All participants understood all the risks and questions involved, and agreed to take part in these surveys voluntarily.

### Measurements and Quality Control

Weight, height and head circumference of all subjects were measured and recorded by two trained anthropometrists in a standardized way. The weight was obtained to the nearest 0.05 kg with lever scale while subjects wore the lightest vest, shorts or underwear. The height was taken to the nearest 0.1 cm as supine length on an examining table for those children under 3 years and as standing height on the stadiometer with bare feet for those at age 3 years and over. For those children under 3 years, the assisting observer stood at the headboard to help position the child’s head so that the crown touched the headboard and a vertical line from the ear canal to the lower border of the eye socket was perpendicular to the horizontal board. For those aged 3 years and over, the back of the head, shoulder blades, buttocks, calves, and heels touched the vertical board. The observer got down to a face-to-face level with the child and positioned the child’s head so that a horizontal line drawn from the ear canal to the lower edge of the eye socket ran parallel to the baseboard. The head circumference was measured to the nearest 0.1 cm using a flexible non-stretchable plastic tape over the most prominent part on the back of the head (occiput) and just above the eyebrows (supraorbital ridges). All the field investigators must participate in rigorous training and pass the examination before starting the survey. Unified measuring tools/instruments were equipped to minimize the influence of measurement errors for each field site. All the measurements were undertaken at least one hour after a meal. Measurement errors were not more than 0.5 cm for height and head circumference or not more than 0.05 kg for weight among measurement groups and between two repeated measurements.

### Smoothing Methods

We constructed the Chinese growth curves for each indicator using the Cole’s LMS method [22], which summarizes the distribution of data by gender and age in term of three spline curves called L, M, and S: the Box-Cox transformation power that converted data to normality and minimizes the skewness of the dataset (L), the median (M), and the coefficient of variation (S). Any required centile curve is defined in terms of the L, M, and S curves as follows:
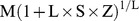
where Z is the Z score corresponding to the required centile (for example, Z = 0 gives the median M or Z = 0.67 gives the 75^th^ centile) and the values of L, M, and S vary with age and gender.

#### Weight-for-age

The single model was fitted to generate one continuous set of curves constituting each gender-specific weight-for-age 0–18 years.

#### Length/height-for-age

The length/height-for-age curve consisted of two parts: a part based on length (length-for-age, 0–36 months) and another on height (height-for-age, 3–18 years), which were constructed using the same model but the final curves reflected the average difference between recumbent length and standing height. To fit a single model in the whole age range, the average difference 0.7 cm (refer to the WHO data [11]) was added to the height values (≧3 years) before merging them with the length data (<3 years). After the model of “length”-for-age was fitted, the length-for-age 0–36 months was directly obtained; the median curve was shifted back downwards by 0.7 cm for ages above 3 years, and the coefficient of variation curve adjusted to the new median values to produce the height-for-age 3–18 years.

#### Head circumference-for-age

Similar to the model of weight, the single LMS model was established based on the NSPGDC data to derive head circumference-for-age 0–6 years for each gender.

#### Weight-for-length/height

The construction of weight-for-length (45 to 110 cm) and weight-for-height (65 to 125 cm) curves followed a similar procedure applied to establish the length/height-for-age curve. To fit a single model, 0.7 cm was added to the height values, and after the model was fitted, the weight-for-“length” curves in the “length” interval 45 to 110 cm were derived directly as the weight-for-length for genders and 65.7 to 125.7 cm were shifted back by 0.7 cm to finally derive the weight-for-height curve corresponding to the height range 65 cm to 125 cm.

#### BMI-for-age

BMI is a ratio with squared length or height in the denominator and weight in the numerator. To address the differences between length and height, the approach used for constructing the BMI-for-age curves was different from that described for length/height-for-age. The two sets of the LMS models based on the length and height values were developed separately. Firstly, the length based BMI-for-age curves were constructed to derive BMI-for-age under 36 months. Secondly, the length data (below 3 years) reduced 0.7 cm was combined to the height data (3–20 years), and then the “height” based BMI-for-age curve were fitted from 0 to 20 years to finally derive BMI-for-age 3–18 years.

### The WHO Standards/References

The WHO standards were primarily based on data collected through the WHO Multicentre Growth Reference Study which was a population-based study conducted in Brazil, Ghana, India, Norway, Oman, and the United States between 1997 and 2003. The study combined a longitudinal follow-up from birth to 24 months with a cross sectional component of children aged 18–71 months. Weight-for-age, length/height-for-age, head circumference-for-age, weight-for-length/height and BMI-for-age Z-score curves were generated for boys and girls aged 0–60 months. The WHO growth references were developed using the same sample as that used for the construction of the original NCHS charts for weight-for-age 5–10 years, height-for-age and BMI-for-age 5–19 years, pooling three data sets [23]. The first and second data sets were from the Health Examination Survey (HES) Cycle II (6–11 years) and Cycle III (12–17 years). The third data set was from the Health and Nutrition Examination Survey (HANES) Cycle I (birth to 74 years), from which only data from the 1 to 24 years age range were used. The full set of tables and charts was available on the WHO website (www.who.int/childgrowth/en).

### Statistic Analysis

LmsChartMaker Pro software 2.3 was used to develop the Chinese growth curves, and the chosen equivalent degrees of freedom (edf) for fitting the L, M and S curves were the weight (boys 051609r, girls 031609r), length/height (031913r, 031813r), head circumference (031104r, 031104r), weight-for-length/height (051109r, 041108r) and BMI (051611t, 0.2, 0, 041611t, 0.2, 0). We compared the empirical −2, 0 and +2 SD curves for weight-for-age, length/height-for-age, head circumference-for-age, weight-for-length/height and BMI -for-age between the China and WHO, respectively.

## Results

### The China References

**Tables 2** and **3** gave weight-for-age and length/height-for-age −2, −1, 0, +1 and +2 SD reference values and L, M and S parameters for Chinese boys and girls from 0 to 18 years at a convenient age interval, and **Table 4** for head circumference-for-age from 0 to 6 years. **Tables 5** and **6** presented weight-for-length (45 to 110 cm) and weight-for-height (65 to 125 cm) at each 5 cm interval. **Tables 7** presented BMI-for-age from 0 to 18 years at a convenient age interval.

**Table 2:** Weight-for-age SD curves (in kg) for Chinese boys and girls, 0–18 years.

Age (Month)*	Boys								Girls							
	L	M	S	−2 SD	−1 SD	Median	+1 SD	+2 SD	L	M	S	−2 SD	−1 SD	Median	+1 SD	+2 SD
0	0.35	3.32	0.1202	2.58	2.93	3.32	3.73	4.18	−0.14	3.21	0.1203	2.54	2.85	3.21	3.63	4.10
1	0.29	4.51	0.1190	3.52	3.99	4.51	5.07	5.67	−0.15	4.20	0.1182	3.33	3.74	4.20	4.74	5.35
2	0.24	5.68	0.1172	4.47	5.05	5.68	6.38	7.14	−0.17	5.21	0.1160	4.15	4.65	5.21	5.86	6.60
3	0.19	6.70	0.1154	5.29	5.97	6.70	7.51	8.40	−0.18	6.13	0.1138	4.90	5.47	6.13	6.87	7.73
4	0.15	7.45	0.1139	5.91	6.64	7.45	8.34	9.32	−0.18	6.83	0.1121	5.48	6.11	6.83	7.65	8.59
5	0.12	8.00	0.1129	6.36	7.14	8.00	8.95	9.99	−0.19	7.36	0.1109	5.92	6.59	7.36	8.23	9.23
6	0.09	8.41	0.1123	6.70	7.51	8.41	9.41	10.50	−0.20	7.77	0.1101	6.26	6.96	7.77	8.68	9.73
8	0.06	9.05	0.1114	7.23	8.09	9.05	10.11	11.29	−0.20	8.41	0.1091	6.79	7.55	8.41	9.39	10.51
10	0.03	9.58	0.1109	7.67	8.58	9.58	10.71	11.95	−−0.21	8.94	0.1084	7.23	8.03	8.94	9.98	11.16
12	0.01	10.05	0.1106	8.06	9.00	10.05	11.23	12.54	−0.22	9.40	0.1080	7.61	8.45	9.40	10.48	11.73
15	−0.01	10.68	0.1103	8.57	9.57	10.68	11.93	13.32	−0.22	10.02	0.1078	8.12	9.01	10.02	11.18	12.50
18	−0.04	11.29	0.1101	9.07	10.12	11.29	12.61	14.09	−0.23	10.65	0.1079	8.63	9.57	10.65	11.88	13.29
21	−0.07	11.93	0.1101	9.59	10.69	11.93	13.33	14.90	−0.24	11.30	0.1084	9.15	10.15	11.30	12.61	14.12
24	−0.09	12.54	0.1101	10.09	11.24	12.54	14.01	15.67	−0.24	11.92	0.1092	9.64	10.70	11.92	13.31	14.92
30	−0.13	13.64	0.1104	10.97	12.22	13.64	15.24	17.06	−0.26	13.05	0.1108	10.52	11.70	13.05	14.60	16.39
36	−0.17	14.65	0.1108	11.79	13.13	14.65	16.39	18.37	−0.27	14.13	0.1123	11.36	12.65	14.13	15.83	17.81
42	−0.21	15.63	0.1117	12.57	14.00	15.63	17.50	19.65	−0.28	15.16	0.1136	12.16	13.55	15.16	17.01	19.17
48	−0.25	16.64	0.1133	13.35	14.88	16.64	18.67	21.01	−0.29	16.17	0.1155	12.93	14.44	16.17	18.19	20.54
54	−0.29	17.75	0.1160	14.18	15.84	17.75	19.98	22.57	−0.30	17.22	0.1181	13.71	15.33	17.22	19.42	22.00
60	−0.33	18.98	0.1201	15.06	16.87	18.98	21.46	24.38	−0.31	18.26	0.1215	14.44	16.20	18.26	20.66	23.50
66	−0.36	20.18	0.1253	15.87	17.85	20.18	22.94	26.24	−0.32	19.33	0.1257	15.18	17.09	19.33	21.98	25.12
72	−0.39	21.26	0.1311	16.56	18.71	21.26	24.32	28.03	−0.33	20.37	0.1301	15.87	17.94	20.37	23.27	26.74
78	−0.41	22.45	0.1385	17.27	19.62	22.45	25.89	30.13	−0.34	21.44	0.1351	16.55	18.78	21.44	24.61	28.46
84	−0.44	24.06	0.1486	18.20	20.83	24.06	28.05	33.08	−0.35	22.64	0.1408	17.31	19.74	22.64	26.16	30.45
90	−0.45	25.72	0.1589	19.11	22.06	25.72	30.33	36.24	−0.36	23.93	0.1469	18.10	20.74	23.93	27.83	32.64
96	−0.45	27.33	0.1687	19.97	23.23	27.33	32.57	39.41	−0.36	25.25	0.1532	18.88	21.75	25.25	29.56	34.94
102	−0.45	28.91	0.1774	20.79	24.37	28.91	34.78	42.54	−0.37	26.67	0.1600	19.71	22.83	26.67	31.45	37.49
108	−0.43	30.46	0.1846	21.62	25.50	30.46	36.92	45.52	−0.38	28.19	0.1675	20.56	23.96	28.19	33.51	40.32
114	−0.40	32.09	0.1906	22.50	26.70	32.09	39.12	48.51	−0.38	29.87	0.1755	21.49	25.21	29.87	35.82	43.54
120	−0.36	33.74	0.1953	23.40	27.93	33.74	41.31	51.38	−0.39	31.76	0.1833	22.54	26.60	31.76	38.41	47.15
126	−0.30	35.58	0.1990	24.43	29.33	35.58	43.69	54.37	−0.39	33.80	0.1893	23.74	28.16	33.80	41.15	50.92
132	−0.24	37.69	0.2016	25.64	30.95	37.69	46.33	57.58	−0.39	36.10	0.1923	25.23	29.99	36.10	44.09	54.78
138	−0.17	39.98	0.2036	26.96	32.73	39.98	49.19	60.96	−0.40	38.40	0.1916	26.89	31.93	38.40	46.87	58.21
144	−0.10	42.49	0.2056	28.41	34.67	42.49	52.31	64.68	−0.41	40.77	0.1873	28.77	34.04	40.77	49.54	61.22
150	−0.06	45.13	0.2063	30.01	36.76	45.13	55.54	68.51	−0.42	42.89	0.1805	30.64	36.04	42.89	51.75	63.44
156	−0.04	48.08	0.2045	32.04	39.22	48.08	59.04	72.60	−0.43	44.79	0.1720	32.50	37.94	44.79	53.55	64.99
162	−0.05	50.85	0.1999	34.22	41.67	50.85	62.16	76.16	−0.44	46.42	0.1631	34.23	39.66	46.42	54.99	66.03
168	−0.10	53.37	0.1928	36.54	44.08	53.37	64.84	79.07	−0.45	47.83	0.1548	35.80	41.18	47.83	56.16	66.77
174	−0.16	55.43	0.1847	38.71	46.20	55.43	66.86	81.11	−0.46	48.97	0.1477	37.13	42.45	48.97	57.06	67.28
180	−0.22	57.08	0.1766	40.63	48.00	57.08	68.35	82.45	−0.47	49.82	0.1422	38.16	43.42	49.82	57.72	67.61
186	−0.28	58.39	0.1692	42.26	49.49	58.39	69.44	83.32	−0.48	50.45	0.1379	38.94	44.15	50.45	58.19	67.82
192	−0.33	59.35	0.1634	43.51	50.62	59.35	70.20	83.85	−0.48	50.81	0.1355	39.39	44.56	50.81	58.45	67.93
198	−0.36	60.12	0.1586	44.54	51.53	60.12	70.79	84.21	−0.48	51.07	0.1337	39.72	44.87	51.07	58.64	68.00
204	−0.39	60.68	0.1550	45.28	52.20	60.68	71.20	84.45	−0.49	51.20	0.1328	39.88	45.01	51.20	58.73	68.04
210	−0.41	61.10	0.1523	45.86	52.71	61.10	71.51	84.61	−0.49	51.31	0.1320	40.02	45.14	51.31	58.81	68.07
216	−0.43	61.40	0.1504	46.27	53.08	61.40	71.73	84.72	−0.49	51.41	0.1313	40.15	45.26	51.41	58.88	68.10

SD, Standard deviation.

* Exact ages not age groups.

**Table 3:** Length/height-for-age SD curves (in cm) for Chinese boys and girls, 0–18 years.

Age (Month)*	Boys								Girls							
	L	M	S	−2SD	−1SD	Median	+1SD	+2SD	L	M	S	−2SD	−1SD	Median	+1SD	+2SD
0	0.61	50.4	0.0352	46.9	48.6	50.4	52.2	54.0	0.45	49.7	0.0344	46.4	48.0	49.7	51.4	53.2
1	0.59	54.8	0.0379	50.7	52.7	54.8	56.9	59.0	0.43	53.7	0.0371	49.8	51.7	53.7	55.7	57.8
2	0.57	58.7	0.0383	54.3	56.5	58.7	61.0	63.3	0.40	57.4	0.0378	53.2	55.3	57.4	59.6	61.8
3	0.55	62.0	0.0369	57.5	59.7	62.0	64.3	66.6	0.38	60.6	0.0365	56.3	58.4	60.6	62.8	65.1
4	0.54	64.6	0.0357	60.1	62.3	64.6	66.9	69.3	0.36	63.1	0.0350	58.8	61.0	63.1	65.4	67.7
5	0.53	66.7	0.0352	62.1	64.4	66.7	69.1	71.5	0.35	65.2	0.0345	60.8	62.9	65.2	67.4	69.8
6	0.52	68.4	0.0351	63.7	66.0	68.4	70.8	73.3	0.33	66.8	0.0346	62.3	64.5	66.8	69.1	71.5
8	0.50	71.2	0.0352	66.3	68.7	71.2	73.7	76.3	0.32	69.6	0.0353	64.8	67.2	69.6	72.1	74.7
10	0.49	74.0	0.0353	68.9	71.4	74.0	76.6	79.3	0.30	72.4	0.0358	67.3	69.8	72.4	75.0	77.7
12	0.48	76.5	0.0358	71.2	73.8	76.5	79.3	82.1	0.29	75.0	0.0360	69.7	72.3	75.0	77.7	80.5
15	0.47	79.8	0.0368	74.0	76.9	79.8	82.8	85.8	0.28	78.5	0.0364	72.9	75.6	78.5	81.4	84.3
18	0.47	82.7	0.0378	76.6	79.6	82.7	85.8	89.1	0.28	81.5	0.0371	75.6	78.5	81.5	84.6	87.7
21	0.46	85.6	0.0389	79.1	82.3	85.6	89.0	92.4	0.28	84.4	0.0383	78.1	81.2	84.4	87.7	91.1
24	0.46	88.5	0.0398	81.6	85.1	88.5	92.1	95.8	0.28	87.2	0.0396	80.5	83.8	87.2	90.7	94.3
30	0.45	93.3	0.0403	85.9	89.6	93.3	97.1	101.0	0.29	92.1	0.0406	84.8	88.4	92.1	95.9	99.8
36	0.45	97.5	0.0394	90.0	93.7	97.5	101.4	105.3	0.31	96.3	0.0394	88.9	92.5	96.3	100.1	104.1
36	0.45	96.8	0.0397	89.3	93.0	96.8	100.7	104.6	0.31	95.6	0.0397	88.2	91.8	95.6	99.4	103.4
42	0.46	100.6	0.0388	93.0	96.7	100.6	104.5	108.6	0.33	99.4	0.0385	91.9	95.6	99.4	103.3	107.2
48	0.46	104.1	0.0385	96.3	100.2	104.1	108.2	112.3	0.36	103.1	0.0382	95.4	99.2	103.1	107.0	111.1
54	0.47	107.7	0.0387	99.5	103.6	107.7	111.9	116.2	0.39	106.7	0.0386	98.7	102.7	106.7	110.9	115.2
60	0.47	111.3	0.0390	102.8	107.0	111.3	115.7	120.1	0.42	110.2	0.0387	101.8	106.0	110.2	114.5	118.9
66	0.49	114.7	0.0391	105.9	110.2	114.7	119.2	123.8	0.45	113.5	0.0389	104.9	109.2	113.5	118.0	122.6
72	0.50	117.7	0.0396	108.6	113.1	117.7	122.4	127.2	0.48	116.6	0.0394	107.6	112.0	116.6	121.2	126.0
78	0.51	120.7	0.0402	111.1	115.8	120.7	125.6	130.5	0.52	119.4	0.0400	110.1	114.7	119.4	124.3	129.2
84	0.53	124.0	0.0409	114.0	119.0	124.0	129.1	134.3	0.55	122.5	0.0407	112.7	117.6	122.5	127.6	132.7
90	0.55	127.1	0.0414	116.8	121.9	127.1	132.4	137.8	0.59	125.6	0.0413	115.4	120.4	125.6	130.8	136.1
96	0.57	130.0	0.0420	119.3	124.6	130.0	135.5	141.1	0.63	128.5	0.0418	117.9	123.1	128.5	133.9	139.4
102	0.59	132.7	0.0426	121.6	127.1	132.7	138.4	144.2	0.67	131.3	0.0424	120.3	125.8	131.3	136.9	142.6
108	0.62	135.4	0.0431	123.9	129.6	135.4	141.2	147.2	0.71	134.1	0.0432	122.6	128.3	134.1	139.9	145.8
114	0.64	137.9	0.0437	126.0	131.9	137.9	144.0	150.1	0.76	137.0	0.0442	125.0	131.0	137.0	143.1	149.2
120	0.67	140.2	0.0442	127.9	134.0	140.2	146.4	152.7	0.81	140.1	0.0450	127.6	133.8	140.1	146.4	152.8
126	0.70	142.6	0.0450	130.0	136.3	142.6	149.1	155.7	0.86	143.3	0.0454	130.3	136.8	143.3	149.8	156.3
132	0.73	145.3	0.0460	132.1	138.7	145.3	152.1	158.9	0.91	146.6	0.0452	133.4	140.0	146.6	153.3	160.0
138	0.78	148.4	0.0473	134.5	141.4	148.4	155.4	162.6	0.96	149.7	0.0442	136.5	143.1	149.7	156.3	162.9
144	0.83	151.9	0.0488	137.2	144.6	151.9	159.4	166.9	1.00	152.4	0.0424	139.5	145.9	152.4	158.8	165.3
150	0.88	155.6	0.0496	140.2	147.9	155.6	163.3	171.1	1.03	154.6	0.0403	142.1	148.4	154.6	160.8	167.1
156	0.94	159.5	0.0487	144.0	151.8	159.5	167.3	175.1	1.06	156.3	0.0385	144.2	150.3	156.3	162.3	168.3
162	0.99	163.0	0.0464	147.9	155.4	163.0	170.5	178.1	1.08	157.6	0.0369	146.0	151.8	157.6	163.4	169.2
168	1.03	165.9	0.0433	151.5	158.7	165.9	173.1	180.2	1.09	158.6	0.0357	147.2	152.9	158.6	164.3	169.9
174	1.06	168.2	0.0405	154.5	161.3	168.2	175.0	181.8	1.10	159.4	0.0349	148.2	153.8	159.4	164.9	170.4
180	1.09	169.8	0.0384	156.7	163.3	169.8	176.3	182.8	1.11	159.8	0.0343	148.8	154.3	159.8	165.3	170.8
186	1.10	171.0	0.0369	158.3	164.7	171.0	177.3	183.6	1.11	160.1	0.0340	149.2	154.7	160.1	165.6	171.0
192	1.11	171.6	0.0362	159.1	165.4	171.6	177.8	184.0	1.11	160.1	0.0340	149.2	154.7	160.1	165.5	171.0
198	1.12	172.1	0.0356	159.7	165.9	172.1	178.2	184.3	1.11	160.2	0.0339	149.3	154.7	160.2	165.6	171.0
204	1.12	172.3	0.0353	160.1	166.3	172.3	178.4	184.5	1.12	160.3	0.0337	149.5	154.9	160.3	165.7	171.1
210	1.13	172.5	0.0350	160.4	166.5	172.5	178.6	184.6	1.12	160.5	0.0336	149.7	155.1	160.5	165.9	171.2
216	1.13	172.7	0.0349	160.5	166.6	172.7	178.7	184.7	1.12	160.6	0.0335	149.8	155.2	160.6	165.9	171.3

Length for children aged 0–36 months and height for children 3–18 years. SD, Standard deviation.

* Exact ages not age groups.

**Table 4:** Head circumference-for-age SD curves for Chinese boys and girls, 0–6 years.

Age (Month)*	Boys								Girls							
	L	M	S	−2 SD	−1 SD	Median	+1 SD	+2 SD	L	M	S	−2 SD	−1 SD	Median	+1 SD	+2 SD
0	1.39	34.5	0.0338	32.1	33.3	34.5	35.7	36.8	1.31	34.0	0.0347	31.6	32.8	34.0	35.2	36.4
1	0.81	36.9	0.0334	34.5	35.7	36.9	38.2	39.4	0.83	36.2	0.0334	33.8	35.0	36.2	37.4	38.6
2	0.31	38.9	0.0328	36.4	37.6	38.9	40.2	41.5	0.37	38.0	0.0323	35.6	36.8	38.0	39.3	40.5
3	−0.04	40.5	0.0322	37.9	39.2	40.5	41.8	43.2	−0.03	39.5	0.0313	37.1	38.3	39.5	40.8	42.1
4	−0.21	41.7	0.0315	39.2	40.4	41.7	43.1	44.5	−0.33	40.7	0.0306	38.3	39.5	40.7	41.9	43.3
5	−0.25	42.7	0.0308	40.2	41.5	42.7	44.1	45.5	−0.51	41.6	0.0300	39.2	40.4	41.6	42.9	44.3
6	−0.22	43.6	0.0303	41.0	42.3	43.6	44.9	46.3	−0.62	42.4	0.0296	40.0	41.2	42.4	43.7	45.1
8	−0.10	44.8	0.0295	42.2	43.5	44.8	46.1	47.5	−0.68	43.6	0.0291	41.2	42.4	43.6	44.9	46.3
10	0.02	45.7	0.0289	43.1	44.4	45.7	47.0	48.4	−0.66	44.5	0.0287	42.1	43.3	44.5	45.8	47.2
12	0.10	46.4	0.0284	43.8	45.1	46.4	47.7	49.1	−0.62	45.1	0.0285	42.7	43.9	45.1	46.5	47.8
15	0.18	47.0	0.0280	44.5	45.7	47.0	48.4	49.7	−0.55	45.8	0.0281	43.4	44.6	45.8	47.2	48.5
18	0.24	47.6	0.0276	45.0	46.3	47.6	48.9	50.2	−0.47	46.4	0.0279	43.9	45.1	46.4	47.7	49.1
21	0.28	48.0	0.0273	45.5	46.7	48.0	49.4	50.7	−0.40	46.9	0.0276	44.4	45.6	46.9	48.2	49.6
24	0.32	48.4	0.0270	45.9	47.1	48.4	49.8	51.1	−0.33	47.3	0.0273	44.8	46.0	47.3	48.6	50.0
30	0.37	49.1	0.0266	46.5	47.8	49.1	50.4	51.7	−0.21	48.0	0.0269	45.5	46.7	48.0	49.3	50.7
36	0.40	49.6	0.0262	47.0	48.3	49.6	50.9	52.2	−0.09	48.5	0.0265	46.0	47.3	48.5	49.8	51.2
42	0.42	49.9	0.0259	47.4	48.7	49.9	51.3	52.6	0.00	49.0	0.0262	46.5	47.7	49.0	50.3	51.6
48	0.44	50.3	0.0256	47.8	49.0	50.3	51.6	52.9	0.08	49.4	0.0259	46.9	48.1	49.4	50.6	52.0
54	0.46	50.6	0.0254	48.1	49.4	50.6	51.9	53.2	0.16	49.7	0.0256	47.2	48.4	49.7	51.0	52.3
60	0.47	51.0	0.0252	48.4	49.7	51.0	52.2	53.6	0.23	50.0	0.0254	47.5	48.7	50.0	51.3	52.6
66	0.48	51.3	0.0249	48.7	50.0	51.3	52.5	53.8	0.29	50.3	0.0252	47.8	49.0	50.3	51.5	52.8
72	0.49	51.5	0.0248	49.0	50.2	51.5	52.8	54.1	0.35	50.5	0.0251	48.0	49.2	50.5	51.8	53.1

SD, Standard deviation.

* Exact ages not age groups.

**Table 5:** Weight-for-length SD curves (in kg) for Chinese boys and girls, 45–110 cm.

Length (cm)*	Boys								Girls							
	L	M	S	−2 SD	−1 SD	Median	+1 SD	+2 SD	L	M	S	−2 SD	−1 SD	Median	+1 SD	+2 SD
45	0.26	2.20	0.0955	1.81	2.00	2.20	2.42	2.65	−0.20	2.33	0.0998	1.91	2.11	2.33	2.57	2.85
50	0.17	3.25	0.0949	2.68	2.95	3.25	3.57	3.91	−0.24	3.29	0.0968	2.72	2.99	3.29	3.63	4.01
55	0.07	4.54	0.0940	3.76	4.14	4.54	4.99	5.48	−0.29	4.54	0.0931	3.79	4.14	4.54	4.99	5.50
60	−0.05	6.06	0.0921	5.05	5.53	6.06	6.65	7.30	−0.34	5.93	0.0889	4.99	5.43	5.93	6.49	7.13
65	−0.18	7.49	0.0894	6.28	6.85	7.49	8.19	8.98	−0.40	7.26	0.0854	6.16	6.68	7.26	7.92	8.67
70	−0.30	8.69	0.0867	7.34	7.98	8.69	9.49	10.38	−0.46	8.41	0.0830	7.16	7.75	8.41	9.15	9.99
75	−0.40	9.72	0.0843	8.25	8.94	9.72	10.59	11.57	−0.51	9.39	0.0813	8.03	8.67	9.39	10.20	11.13
80	−0.50	10.71	0.0820	9.15	9.88	10.71	11.64	12.70	−0.56	10.34	0.0797	8.88	9.57	10.34	11.22	12.22
85	−0.60	11.75	0.0796	10.09	10.87	11.75	12.74	13.89	−0.61	11.37	0.0785	9.79	10.53	11.37	12.32	13.41
90	−0.70	12.83	0.0775	11.08	11.90	12.83	13.90	15.12	−0.67	12.50	0.0778	10.78	11.58	12.50	13.54	14.73
95	−0.80	13.98	0.0758	12.12	12.99	13.98	15.12	16.44	−0.73	13.75	0.0777	11.87	12.75	13.75	14.90	16.22
100	−0.91	15.27	0.0753	13.26	14.20	15.27	16.51	17.96	−0.80	15.09	0.0787	13.01	13.98	15.09	16.37	17.86
105	−1.02	16.71	0.0769	14.49	15.52	16.71	18.11	19.76	−0.86	16.48	0.0811	14.16	15.24	16.48	17.93	19.63
110	−1.12	18.27	0.0811	15.74	16.91	18.27	19.89	21.85	−0.92	17.96	0.0848	15.34	16.55	17.96	19.62	21.60

SD, Standard deviation.

* Exact length not length groups.

**Table 6:** Weight-for-height SD curves (in kg) for Chinese boys and girls, 65–125 cm.

Height (cm)*	Boys								Girls							
	L	M	S	−2 SD	−1 SD	Median	+1 SD	+2 SD	L	M	S	−2 SD	−1 SD	Median	+1 SD	+2 SD
65	−0.20	7.67	0.0890	6.44	7.02	7.67	8.39	9.19	−0.41	7.43	0.0850	6.31	6.84	7.43	8.11	8.87
70	−0.31	8.84	0.0863	7.47	8.12	8.84	9.65	10.56	−0.47	8.55	0.0827	7.29	7.89	8.55	9.31	10.16
75	−0.41	9.85	0.0840	8.38	9.07	9.85	10.73	11.73	−0.52	9.52	0.0810	8.15	8.79	9.52	10.34	11.28
80	−0.51	10.85	0.0816	9.27	10.02	10.85	11.79	12.87	−0.56	10.48	0.0795	9.00	9.70	10.48	11.37	12.38
85	−0.61	11.89	0.0793	10.22	11.01	11.89	12.90	14.06	−0.62	11.52	0.0784	9.92	10.67	11.52	12.48	13.58
90	−0.71	12.99	0.0773	11.22	12.05	12.99	14.06	15.30	−0.68	12.66	0.0778	10.92	11.74	12.66	13.72	14.93
95	−0.82	14.15	0.0757	12.27	13.15	14.15	15.30	16.64	−0.74	13.94	0.0778	12.03	12.92	13.94	15.10	16.44
100	−0.93	15.46	0.0754	13.43	14.38	15.46	16.72	18.19	−0.80	15.28	0.0790	13.17	14.16	15.28	16.58	18.10
105	−1.04	16.92	0.0773	14.66	15.71	16.92	18.34	20.03	−0.87	16.68	0.0815	14.32	15.42	16.68	18.15	19.89
110	−1.13	18.50	0.0818	15.92	17.11	18.50	20.16	22.18	−0.93	18.18	0.0855	15.51	16.74	18.18	19.87	21.90
115	−1.21	20.28	0.0890	17.26	18.63	20.28	22.28	24.77	−1.00	19.84	0.0907	16.80	18.19	19.84	21.82	24.24
120	−1.27	22.30	0.0988	18.69	20.31	22.30	24.78	27.99	−1.07	21.71	0.0971	18.20	19.79	21.71	24.05	26.99
125	−1.31	24.64	0.1110	20.28	22.21	24.64	27.78	32.04	−1.16	23.79	0.1041	19.74	21.57	23.79	26.59	30.20

SD, Standard deviation.

* Exact height not height groups.

**Table 7:** BMI-for-age SD curves (in kg/m^2^) for Chinese boys and girls, 0–18 years.

Age (Month)*	Boys								Girls							
	L	M	S	−2SD	−1SD	Median	+1SD	+2SD	L	M	S	−2SD	−1SD	Median	+1SD	+2SD
0	−0.03	13.07	0.0837	11.06	12.02	13.07	14.21	15.45	−0.44	13.00	0.0878	10.98	11.93	13.00	14.22	15.61
1	0.72	14.91	0.0861	12.41	13.64	14.91	16.21	17.54	−0.31	14.52	0.0844	12.31	13.36	14.52	15.81	17.26
2	0.47	16.48	0.0860	13.77	15.10	16.48	17.93	19.44	−0.34	15.84	0.0848	13.43	14.57	15.84	17.27	18.87
3	0.25	17.48	0.0866	14.64	16.02	17.48	19.04	20.71	−0.37	16.69	0.0850	14.16	15.35	16.69	18.20	19.90
4	0.07	17.87	0.0874	14.99	16.37	17.87	19.50	21.27	−0.40	17.14	0.0848	14.55	15.77	17.14	18.69	20.43
5	−0.08	17.97	0.0878	15.10	16.47	17.97	19.63	21.45	−0.43	17.35	0.0843	14.74	15.97	17.35	18.90	20.66
6	−0.19	17.96	0.0875	15.12	16.47	17.96	19.62	21.47	−0.45	17.41	0.0835	14.82	16.04	17.41	18.96	20.71
8	−0.36	17.76	0.0856	15.04	16.32	17.76	19.37	21.19	−0.50	17.31	0.0818	14.79	15.98	17.31	18.82	20.53
10	−0.47	17.48	0.0833	14.89	16.11	17.48	19.03	20.79	−0.54	17.05	0.0802	14.62	15.77	17.05	18.51	20.17
12	−0.54	17.19	0.0814	14.71	15.87	17.19	18.68	20.38	−0.57	16.74	0.0790	14.39	15.50	16.74	18.15	19.76
15	−0.60	16.78	0.0797	14.41	15.52	16.78	18.20	19.83	−0.62	16.32	0.0779	14.06	15.12	16.32	17.67	19.22
18	−0.64	16.47	0.0787	14.18	15.26	16.47	17.86	19.45	−0.66	16.03	0.0775	13.83	14.86	16.03	17.36	18.88
21	−0.68	16.26	0.0779	14.03	15.08	16.26	17.62	19.18	−0.70	15.84	0.0773	13.68	14.69	15.84	17.15	18.66
24	−0.73	16.07	0.0770	13.88	14.91	16.07	17.39	18.92	−0.73	15.67	0.0772	13.54	14.54	15.67	16.97	18.46
30	−0.85	15.73	0.0754	13.64	14.62	15.73	17.00	18.48	−0.79	15.40	0.0769	13.32	14.30	15.40	16.68	18.15
36	−0.97	15.43	0.0746	13.43	14.36	15.43	16.68	18.14	−0.84	15.25	0.0771	13.19	14.15	15.25	16.52	17.99
36	−1.02	15.66	0.0745	13.63	14.57	15.66	16.92	18.41	−0.95	15.42	0.0773	13.35	14.31	15.42	16.70	18.22
42	−1.09	15.45	0.0752	13.44	14.37	15.45	16.71	18.21	−0.98	15.27	0.0782	13.20	14.16	15.27	16.56	18.10
48	−1.14	15.32	0.0767	13.30	14.23	15.32	16.60	18.13	−1.01	15.15	0.0801	13.06	14.03	15.15	16.47	18.05
54	−1.17	15.23	0.0790	13.18	14.12	15.23	16.55	18.14	−1.03	15.06	0.0826	12.93	13.92	15.06	16.42	18.05
60	−1.19	15.22	0.0823	13.10	14.07	15.22	16.60	18.28	−1.05	14.99	0.0854	12.81	13.82	14.99	16.40	18.10
66	−1.19	15.27	0.0866	13.05	14.06	15.27	16.73	18.54	−1.06	14.96	0.0884	12.72	13.75	14.96	16.42	18.20
72	−1.19	15.35	0.0916	13.00	14.07	15.35	16.91	18.87	−1.06	14.96	0.0915	12.66	13.71	14.96	16.47	18.34
78	−1.17	15.45	0.0971	12.97	14.09	15.45	17.13	19.26	−1.07	14.97	0.0949	12.60	13.68	14.97	16.55	18.51
84	−1.15	15.59	0.1027	12.97	14.15	15.59	17.40	19.72	−1.07	15.02	0.0984	12.56	13.67	15.02	16.66	18.73
90	−1.13	15.77	0.1084	12.99	14.24	15.77	17.70	20.22	−1.07	15.10	0.1022	12.55	13.70	15.10	16.82	19.01
96	−1.11	15.96	0.1140	13.03	14.34	15.96	18.03	20.76	−1.07	15.21	0.1062	12.56	13.76	15.21	17.03	19.36
102	−1.08	16.18	0.1192	13.09	14.47	16.18	18.39	21.32	−1.06	15.37	0.1103	12.61	13.85	15.37	17.29	19.77
108	−1.06	16.42	0.1240	13.18	14.62	16.42	18.76	21.90	−1.06	15.57	0.1142	12.69	13.98	15.57	17.59	20.23
114	−1.03	16.68	0.1283	13.28	14.79	16.68	19.14	22.48	−1.06	15.82	0.1180	12.81	14.15	15.82	17.94	20.75
120	−1.01	16.96	0.1321	13.42	14.98	16.96	19.54	23.06	−1.06	16.09	0.1215	12.97	14.36	16.09	18.33	21.31
126	−0.99	17.24	0.1353	13.57	15.19	17.24	19.94	23.62	−1.06	16.40	0.1245	13.15	14.59	16.40	18.75	21.90
132	−0.97	17.54	0.1379	13.73	15.41	17.54	20.33	24.17	−1.05	16.74	0.1270	13.36	14.86	16.74	19.18	22.49
138	−0.95	17.83	0.1401	13.91	15.63	17.83	20.72	24.70	−1.05	17.09	0.1290	13.60	15.14	17.09	19.63	23.09
144	−0.94	18.12	0.1419	14.09	15.86	18.12	21.09	25.20	−1.05	17.45	0.1305	13.85	15.44	17.45	20.08	23.67
150	−0.93	18.40	0.1433	14.27	16.08	18.40	21.45	25.67	−1.05	17.80	0.1317	14.11	15.74	17.80	20.51	24.23
156	−0.92	18.67	0.1443	14.45	16.30	18.67	21.80	26.11	−1.05	18.15	0.1325	14.37	16.03	18.15	20.93	24.76
162	−0.91	18.94	0.1451	14.64	16.52	18.94	22.12	26.52	−1.05	18.48	0.1331	14.61	16.31	18.48	21.33	25.24
168	−0.90	19.19	0.1457	14.81	16.74	19.19	22.43	26.90	−1.05	18.78	0.1334	14.84	16.58	18.78	21.69	25.69
174	−0.89	19.43	0.1461	14.99	16.94	19.43	22.73	27.25	−1.05	19.06	0.1336	15.06	16.82	19.06	22.01	26.08
180	−0.88	19.66	0.1463	15.16	17.14	19.66	23.00	27.58	−1.05	19.31	0.1337	15.25	17.04	19.31	22.30	26.42
186	−0.87	19.88	0.1464	15.32	17.32	19.88	23.26	27.89	−1.05	19.53	0.1337	15.42	17.23	19.53	22.55	26.72
192	−0.87	20.09	0.1465	15.48	17.50	20.09	23.50	28.17	−1.05	19.72	0.1337	15.58	17.40	19.72	22.77	26.98
198	−0.86	20.29	0.1465	15.63	17.68	20.29	23.73	28.44	−1.05	19.89	0.1336	15.71	17.55	19.89	22.97	27.21
204	−0.85	20.48	0.1464	15.77	17.84	20.48	23.95	28.69	−1.05	20.04	0.1335	15.84	17.69	20.04	23.14	27.41
210	−0.85	20.67	0.1464	15.91	18.00	20.67	24.16	28.93	−1.05	20.18	0.1334	15.95	17.81	20.18	23.30	27.59
216	−0.84	20.84	0.1463	16.04	18.16	20.84	24.36	29.16	−1.05	20.32	0.1333	16.06	17.93	20.32	23.45	27.77

Length based BMI-for-age 0–36 months and height based BMI 3–18 years. SD, Standard deviation.

* Exact age not age groups.

### Comparison of the China and WHO Curves

#### Weight-for-age

**Figure 2:**
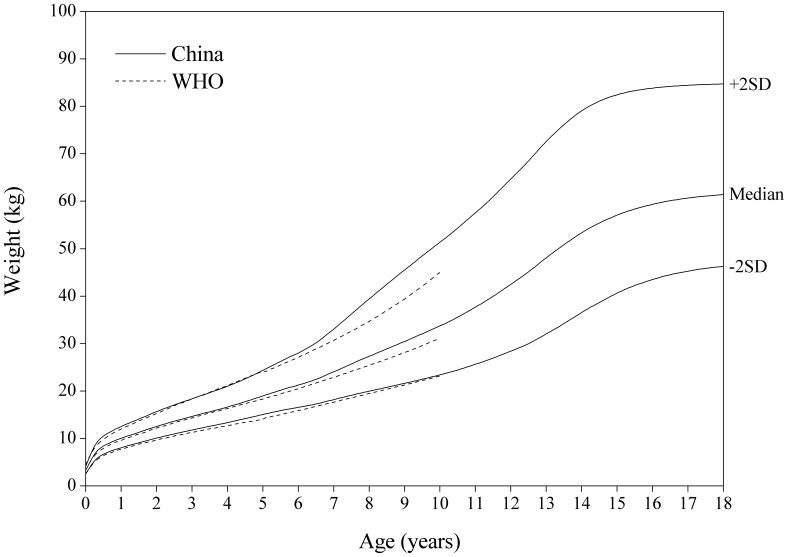
Comparison of the China and WHO weight-for-age SD curves for boys.

**Fig. 2** **,** **3** showed the comparison of the China and WHO weight-for-age SD curves. The Chinese boys are appreciably heavier than the girls. The Chinese boys are strikingly heavier than those in the WHO on the median and +2 SD curves between 6 and 10 years. No significant differences were found between the two for girls.

**Figure 3:**
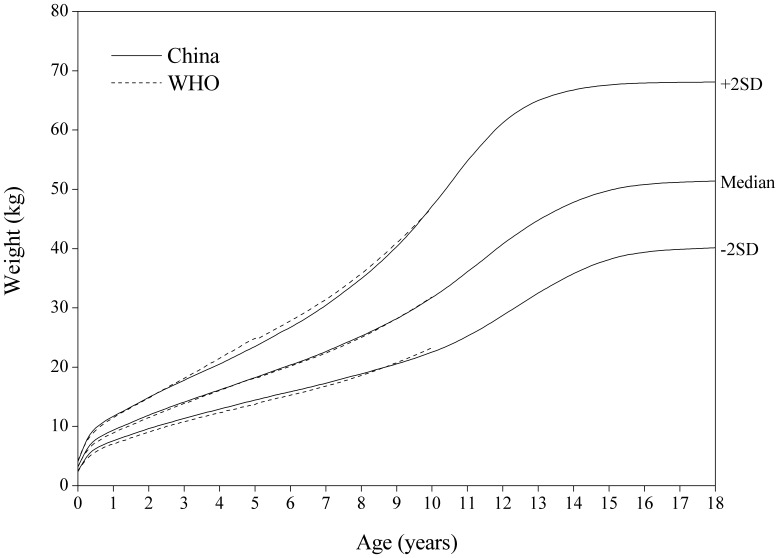
Comparison of the China and WHO weight-for-age SD curves for girls.

#### Length/height-for-age

**Figure 4:**
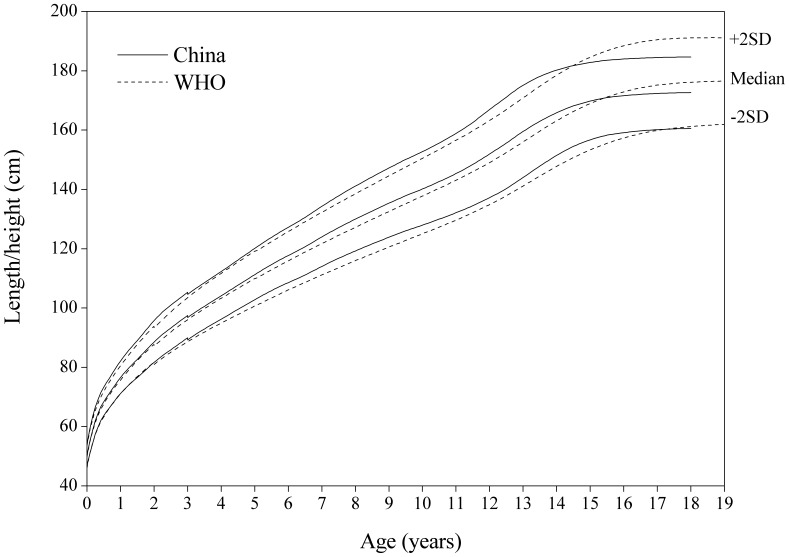
Comparison of the China and WHO length/height-for-age SD curves for boys. Length for boys 0–3 years and height 3–18 for China, and length 0–2 years and height 2–19 for WHO.

**Fig. 4** **,** **5** showed the comparison of the China and WHO length/height-for-age SD curves. On the whole, the China sample is taller than the WHO for boys under 15 years and for girls under 13 years, but is significantly lower for boys over 15 years and girls over 13.

**Figure 5:**
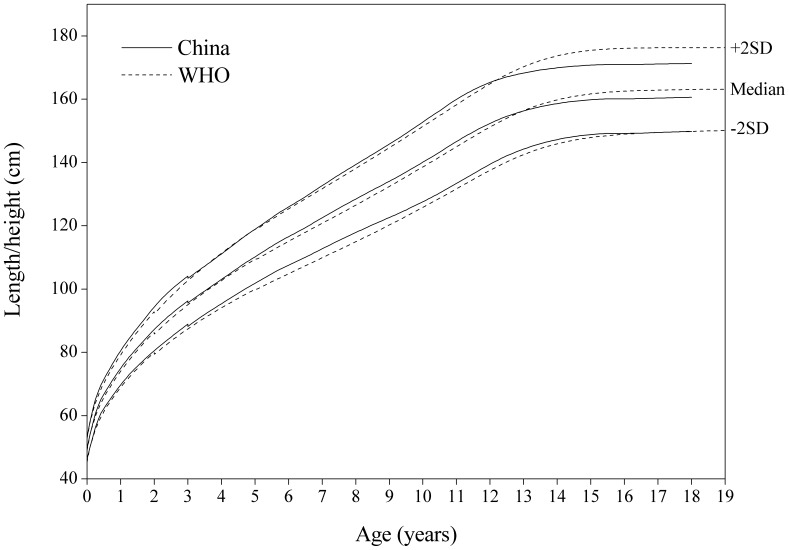
Comparison of the China and WHO length/height-for-age SD curves for girls. Length for girls 0–3 years and height 3–18 for China, and length 0–2 years and height 2–19 for WHO.

#### Head circumference-for-age

**Figure 6:**
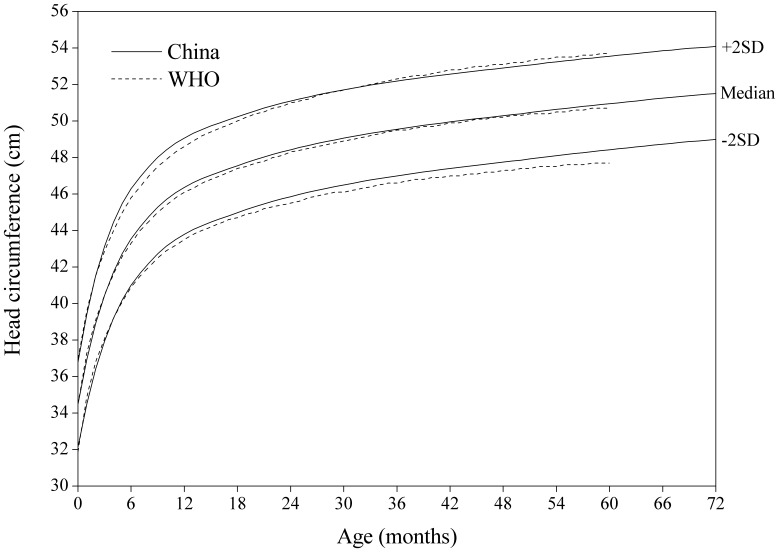
Comparison of the China and WHO head circumference-for-age SD curves for boys.

**Fig. 6** **,** **7** showed that there was no obvious difference in head circumference-for-age SD curves between the China and WHO in the first year, and after the age of one year, the Chinese are slightly higher than those in the WHO on the −2 SD curve.

**Figure 7:**
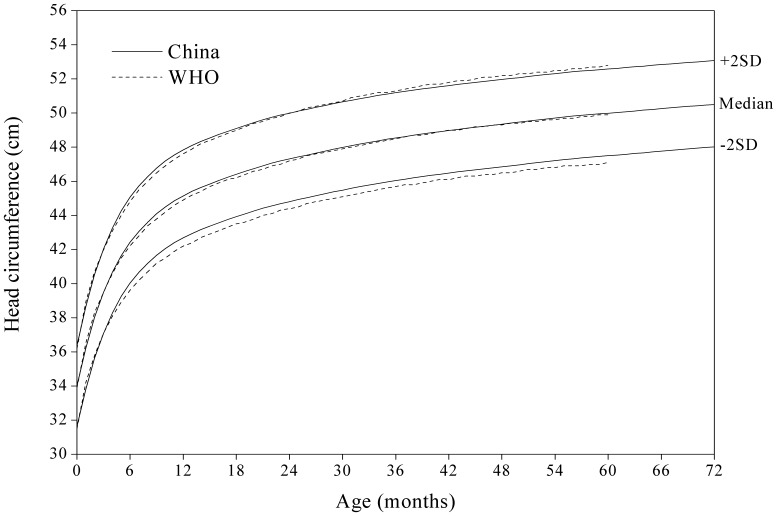
Comparison of the China and WHO head circumference-for-age SD curves for girls.

#### Weight-for-length/height

**Figure 8:**
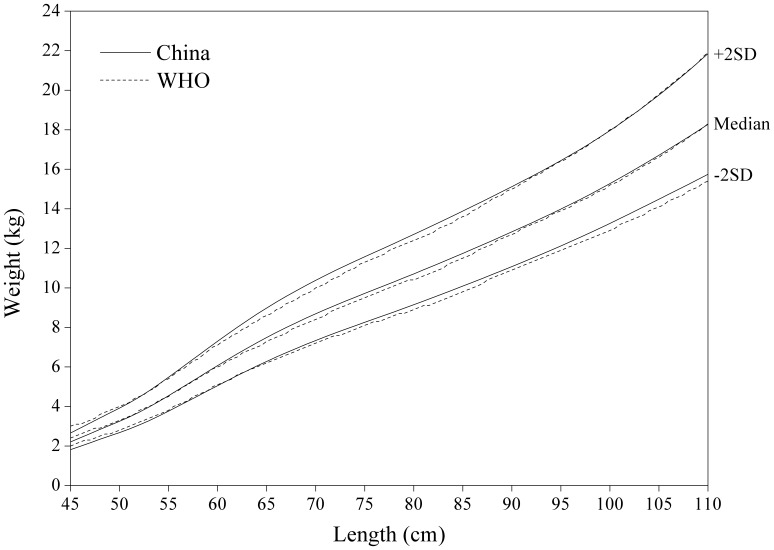
Comparison of the China and WHO weight-for-length SD curves for boys.

**Fig. 8** **,** **9** **,** **10** **,** **11** for weight-for-length/weight displayed fairly small differences between China and WHO. The Chinese girls are generally lighter at height >105 cm, especially on +2 SD curve.

**Figure 9:**
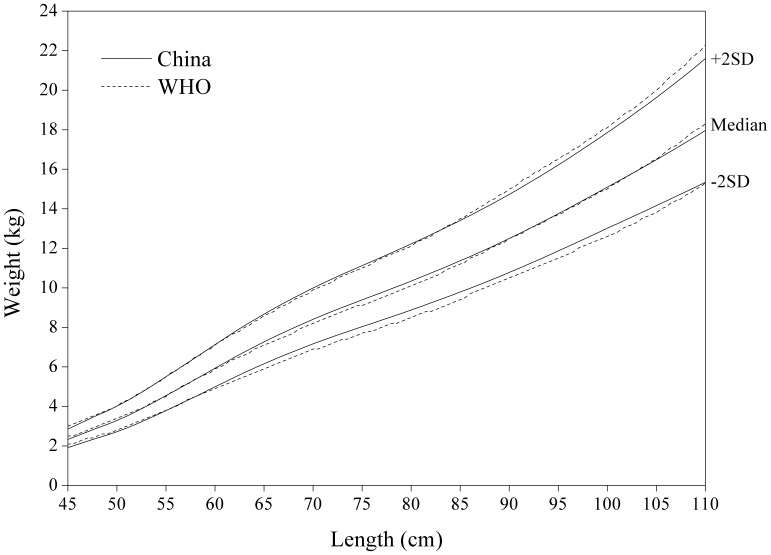
Comparison of the China and WHO weight-for-length SD curves for girls.

**Figure 10:**
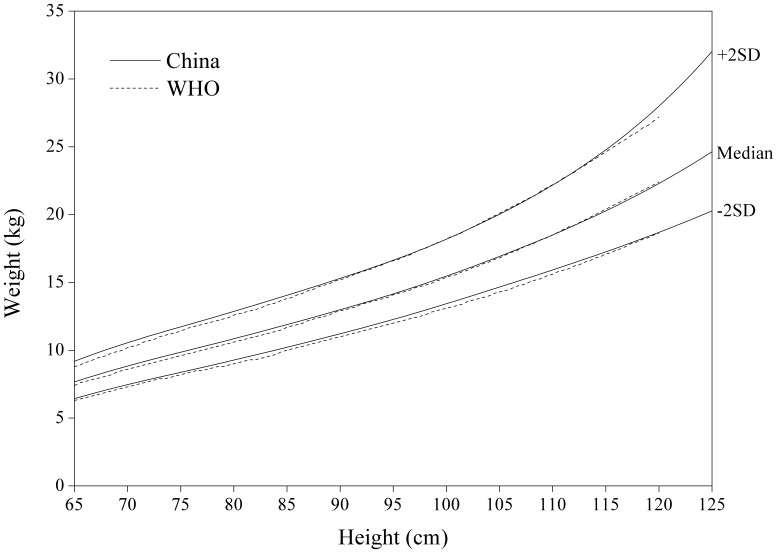
Comparison of the China and WHO weight-for-height SD curves for boys.

**Figure 11:**
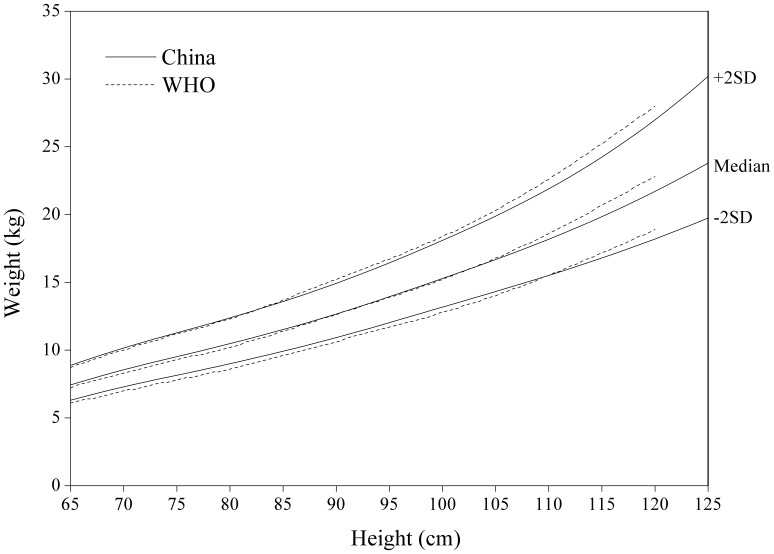
Comparison of the China and WHO weight-for-height SD curves for girls.

#### BMI-for-age

**Figure 12:**
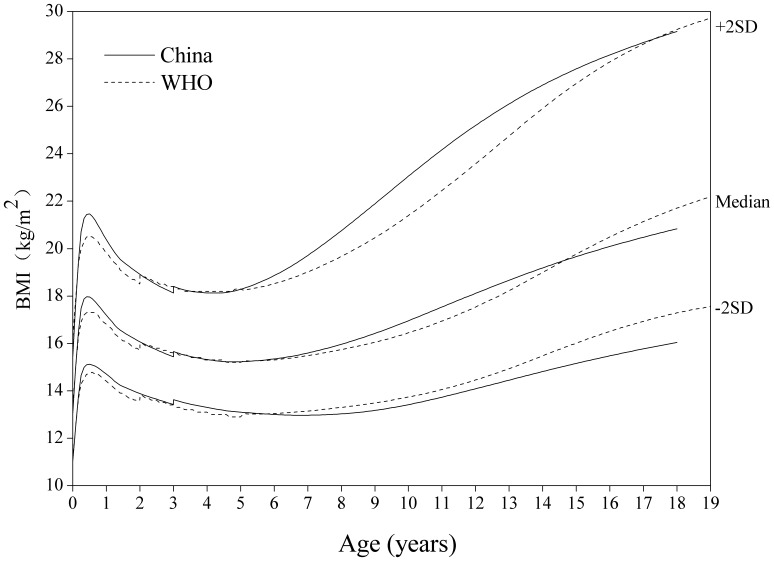
Comparison of the China and WHO BMI-for-age SD curves for boys. Length based BMI for boys 0–3 years and height 3–18 for China, and length based BMI 0–2 years and height 2–19 for WHO.

**Fig. 12** **,** **13** for BMI-for-age displayed notable differences between the two sets of curves, and the boys are very different from the girls. The performance partly reflects the nutritional status of the respective reference populations and might also be a smoothing effect. The Chinese are higher at age around 6 months. The boys’ BMI is generally higher than that of the WHO from 6 to 16 years on the median and +2 SD curve. The girls’ BMI is appreciably lower than that of WHO from 3 to 18 years on the −2, 0 and +2 SD curve.

**Figure 13:**
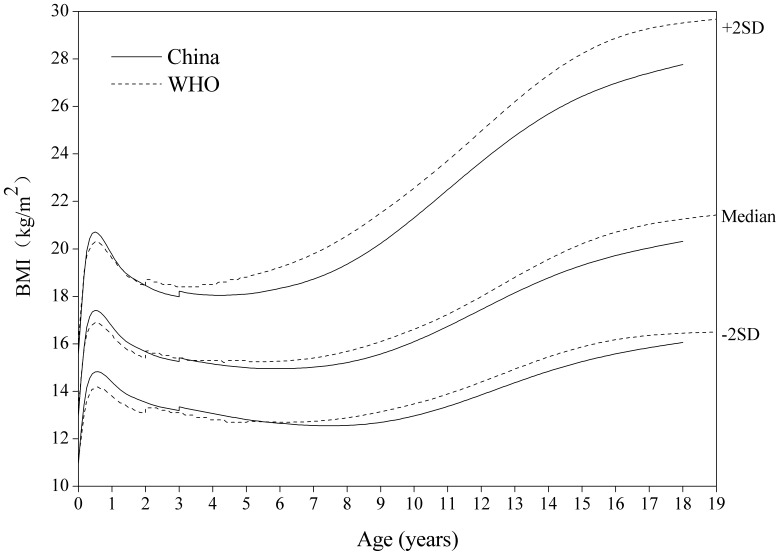
Comparison of the China and WHO BMI-for-age SD curves for girls. Length based BMI for girls 0–3 years and height 3–18 for China, and length based BMI 0–2 years and height 2–19 for WHO.

## Discussion

For many years, the growth reference data of Chinese children and adolescents was obtained from the two series of national surveys, the NSPGDC for preschool children aged 0–7 years and the CNSSCH for school children aged 7–18. These data was routinely updated every 10 years under the secular trends. However, the practical application of those raw reference data in growth monitoring and nutrition assessment was limited, due to the lack of convenient age intervals, the shortage of smoothed percentiles and SD curves, the absence of continuity at the entire age range (separate at age 6–7 years) and the incompleteness of indicators. Therefore, it was necessary to construct a standardized growth curves for Chinese children and adolescents in new century, based on up-to-date national data using the international smoothing method.

The rapid positive secular growth trends will continue in China as the rapid socio-economic development going on [9]. The growth level of urban children was successively taller than suburban ones [24]. The urban data has been widely used as the most representative reference values in many fields since 1975. So, we merged the data from the 4^th^ NSPGDC (0–7 years) with the 5^th^ CNSSCH (6–20 years) into a complete age group to generate the growth curves from birth to 18 years. The reference sample was urban children and adolescents which were representative of healthy, relatively well-nourished population who were born and raised under good conditions with the high living standards and education level in China. In the sample population, the proportion of receiving high school education and above was 84.4% for their fathers and 80.6% for mothers [20] and the overall breast-feeding rate was 85.1% for infants below 4 months and exclusive breast-feeding 47.7% [25].

The two comments of the advisory panels benefited the construction of the China references. The first was that data from the corresponding 9 provinces/cities of the 5^th^ CNSSCH was naturally selected for merging with data from the 4^th^ NSPGDC because those regions were nearly the same geographical positions and relative high socioeconomic background as the 9 cities of the latter. The second was that urban children were selected as the reference sample to develop the curves due to the differences of growth among urban, suburban and rural areas and the growth potential of Chinese children under the rapid secular trends.

Human growth differs throughout history. Data from the Chinese surveys showed the rapid secular growth trends during the past decades [9], [19]. The positive trends reflected the great socioeconomic development after the economic reform and opening up. It had effected greatly the construction and application of the China references. The previous reference data, national or local, had obvious historical limitations in evaluating current performance of growth and nutrition of children and adolescents. The China references provided by this study were the updated standardized national reference values under the positive secular trends and could truly estimate the level of growth and nutrition in current stage.

Human growth differs among the various ethnic groups. The differences of the standards/references among regions or countries were largely caused by the respective reference populations. They might partly be attributed to the study designs (e.g. exclusion criteria, measurement tools and age group intervals) and the smoothing methods. Growth pattern was similar in different ethnic populations, but the differences of the standards/references could not be neglected in practical application, especially for the assessment of individual child. Followings are the main differences and interpretations between China and WHO founded in this study.

The gender difference was very obvious. Chinese boys were appreciably heavier than girls at counterpart age, which was also funded in other studies [10], [16]. For weight and weight related indicators, the nutrition level of Chinese girls was generally lower than that of Western countries, but usually not for boys. For example, boys’ BMI at age 6–16 years in China was higher than their counterparts in the WHO, but girls’ BMI 3–18 years was lower, especially on the +2 SD curve. The reversal performance was also observed in overweight and obesity studies among Chinese children, i.e. the prevalence rates for boys significantly higher than girls’ [10], [26]. Another study, data not from the two series of surveys, also showed the same gender performance [16]. Compared with the Western, the gender-specific performance was an interesting fact, which may be associated with a bigger percentage of the “fat” population in boys and the traditional Chinese cultural and social values. The trend analysis of overweight and obesity in Chinese students during 1985–2010 showed the prevalence rates in boys were higher than those in girls in urban and rural areas [27]. In other words, the “fat” population has a bigger percentage in boys than in girls. In the traditional Chinese culture, boys were expected to be tall and strong so that they can be the major labor force of a family when they grow up. On the opposite, girls were expected to be slim and elegant. If they were too fatty and strong, it may have negative effect of their marriage in the future. So girls and their family may choose to select and limit their daily diet.

Final heights of Chinese populations are significantly shorter than that of the WHO (i.e. U.S. samples) in males and females. The Chinese are taller until adolescence, but then being shorter as adults. This cross-over performance reflects another obvious ethnic characteristic. The taller performance before puberty may be related to the rapid increasing growth trends in the past decades and also the earlier and earlier onset age of puberty, such as earlier 0.2 year per decade between 1985 and 2005 [21]. After 15-year-old for boys and 13-year for girls, the growth sharply slowed down and the average residual growth potential was about 3–4 cm. Their height was close to adult height in boys after 16-year and in girls after 15-year. The varying performance over age among different regions or countries strongly supports the necessity of establishing population-specific references.

The differences in boundary curves (e.g. −2 and +2 SD) are also interesting for each indicator. The Chinese boys are fatter than the WHO for BMI at nearly all ages (with the exception of ages 4–6 where the curves cross over) and later adolescence. The +2 SD curves for the Chinese and WHO cross at age 17, showing that they reach the same BMI but following quite different routes to get there. The Chinese girls are heavier than the WHO’s in early life, particularly on the lower centiles, but the age at adiposity rebound is appreciably later and the later curves are much lower. Cole & Lobstein [28] described the cross-over pattern of WHO that was relatively low in early life and relatively high later as a feature of the WHO standard/reference. De Onis et al [29] suggested the performance of BMI partly reflected the nutrition status of sample and probably there were also some edge effects by comparison of the WHO standards and the CDC 2000 charts. The differences of BMI reflected data sources used by the Chinese and the WHO. Thus, estimates of overweight/obesity might be somewhat different for genders and age groups using the empirical “cut-off points” of the different standards/references.

The disparity of estimated prevalence rates in shortness, underweight, and overweight/obesity might be the differences of boundary centiles between China and WHO. A study written by Mei et al [30] showed estimates of prevalence of key descriptors of growth varied by the chart used and the cutoff values applied which suggested the charts and cutoff values would be a change for office practice. Therefore, for practitioners, the WHO standards might be a better tool for the purpose of comparisons among countries/regions. More attention should be given to further explore the variation of growth among race/ethnicity groups and the practical applications in clinic and public health.

China is a big country, stretching from north latitude 4° to 53° and east longitude 73° to 135° with the disparity of the regional socioeconomics in current stage. The genetic growth potential may vary among different geographical regions and socioeconomic classes. The series of the NSPGDC shows that no sufficient evidence supported the regional growth difference in inter-province/city was reducing [9]. Urban-suburban-rural difference had a narrowing trend but the gap did not disappear according to the systematic analysis of the NSPGDC and another national survey undertaken in rural areas [9], [24]. The series of the CNSSCH displays the height growth rate of Chinese students is influenced by the geography and natural environment. The growth level in eastern China is higher than central-western, successively higher than southern [9]. And the growth level in northern is higher than that in southern [31]. So, from the perspective of socioeconomic classes, this new China references are a “current” reference for urban children and may be an “ideal” standard for suburban and rural children. From the geographic location, the China references are “current” for northern and eastern and may be an “ideal” for central-western and southern.The new China references are the first, multi-indicators, complete age range, smoothed growth reference values which will benefit more than 300 million Chinese children and millions of children of Chinese origins. The present study strongly suggests the China references are obviously advantageous in monitoring and assessing current performance of growth and nutrition of Chinese children and adolescents when compared with other standards or references, especially for individual growth monitoring in clinical practice. The set of references should help to promote the standardization assessment of growth and nutrition throughout the entire childhood in China.
